# Is There Seasonality in Hypothyroidism? A Google Trends Pilot Study

**DOI:** 10.7759/cureus.3965

**Published:** 2019-01-25

**Authors:** Ioannis Ilias, Maria Alexiou, Georgios Meristoudis

**Affiliations:** 1 Internal Medicine, Elena Venizelou Hospital, Athens, GRC; 2 Internal Medicine, Kent and Canterbury Hospital, Canterbury, GBR; 3 Nuclear Medicine, Hippokration Hospital, Thessaloniki, GRC

**Keywords:** data collection, methods, trends, medical informatics, hypothyroidism, epidemiology

## Abstract

Introduction: There may be seasonality in thyroid diseases and internet search data may provide information on disease patterns. In this study we used data from internet searches on hypothyroidism to assess seasonality in this disease.

Methods: We collected worldwide data, as well as data for countries in the southern hemisphere (Brazil, South Africa, and Australia), covering 15 years, from Google Trends with the search term “hypothyroidism+thyroiditis (the commonest cause of hypothyroidism)” and “fatigue+weakness (the commonest symptoms of hypothyroidism)”. We looked for periodicity in relevant internet searches by calculating autocorrelations; we also looked at the cross-correlation of internet searches for “hypothyroidism+thyroiditis” and “fatigue+weakness” and we compared the results by season with the Kruskall-Wallis test.

Results: There was periodicity in the relevant internet searches and strong cross-correlations between internet searches for “hypothyroidism+thyroiditis” and “fatigue+weakness” worldwide and for South Africa and Australia. In both the northern and the southern hemispheres there were significantly more hypothyroidism-related internet searches during spring (p<0.05).

Conclusion: Hypothyroidism was more popular in internet searches at springtime in the northern and the southern hemispheres. Thus, although this analysis is coarse, it seems that some seasonality can be inferred on hypothyroidism, taking into account the limitations of our approach.

## Introduction

Seasonality has been reported in subclinical hypothyroidism [1], subacute thyroiditis [2], and thyroid function tests [3]. Internet users perform internet searches, often to excess, triggered by personal symptoms or diseases. Internet search data may provide information - albeit indirectly - on disease patterns [4]. Recently, temporal and geographic correlation was noted between Google Trends queries and coronary artery disease prevalence [5]. In this pilot/observational study we used data from Google’s internet searches (where 76% of internet searches originate [6]) on hypothyroidism to assess seasonality in this disease.

## Materials and methods

We collected data from Google Trends regarding relative [internet] search volumes (RSVs) with the search terms “hypothyroidism+thyroiditis (the commonest cause of hypothyroidism [7])” and “fatigue+weakness (these being among the commonest - albeit nonspecific - hypothyroidism symptoms [7, 8])” (Google does not report search volumes, in lieu of presenting RSVs, which are percentages relative to the peak search volume obtained during the specified time period and scaled by the total search volume for each specific search term. Thus these numbers do not represent absolute search volume numbers; the data are normalized and presented on a scale from zero to 100 [9]). We collected data worldwide from January 1, 2004 to December 31, 2018 (with the search terms in English) as well as data from countries in the southern hemisphere with large relevant search volumes: Brazil (in Portuguese), South Africa and Australia (in English). Analysis was done following procedures already delineated in the literature [10]. More in detail, we calculated autocorrelation functions (ACFs) for the RSVs with the Durbin-Watson test and we also calculated the cross-correlations between RSVs for “hypothyroidism+thyroiditis” and “fatigue+weakness” following smoothing of the data with Winter’s method and implementation of an autoregressive integrated moving average (ARIMA) model. Comparisons of RSVs by meteorological season (winter, spring, summer, and autumn) were done with the Kruskall-Wallis test. Statistical significance was set at p<0.05 (regarding cross-correlation, a coefficient with an absolute value of at least 0.15 at zero lag was considered to be significant at the 0.05 level [11]).

## Results

**Figure 1:**
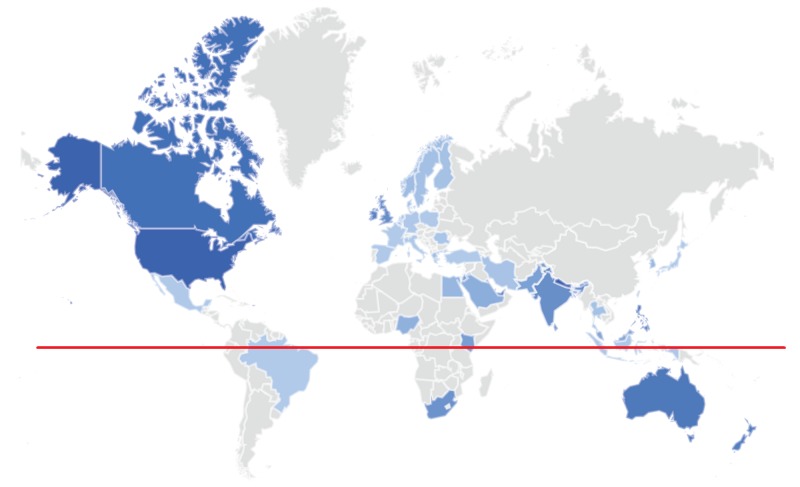
Graphic map of search term popularity for “hypothyroidism+thyroiditis” by location (worldwide). Color intensity is relative to the total number of Google searches performed during the study’s time span. The highest (100%) popularity was noted in Nepal, with 88% in the USA, 45% in the UK, 43% in South Africa and 63% in Australia. *The red line indicates the equator.*

**Figure 2:**
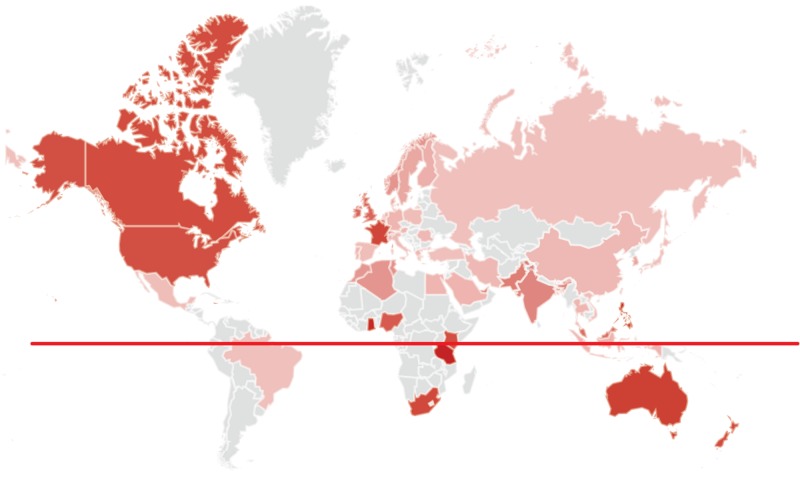
Graphic map of search term popularity for “fatigue+weakness” by location (worldwide). Color intensity is relative to the total number of Google searches performed during the study’s time span. The highest (100%) popularity was noted in Jamaica, with 67% in the USA, 52% in the UK, 70% in South Africa and 74% in Australia. *The red line indicates the equator.*

**Figure 3:**
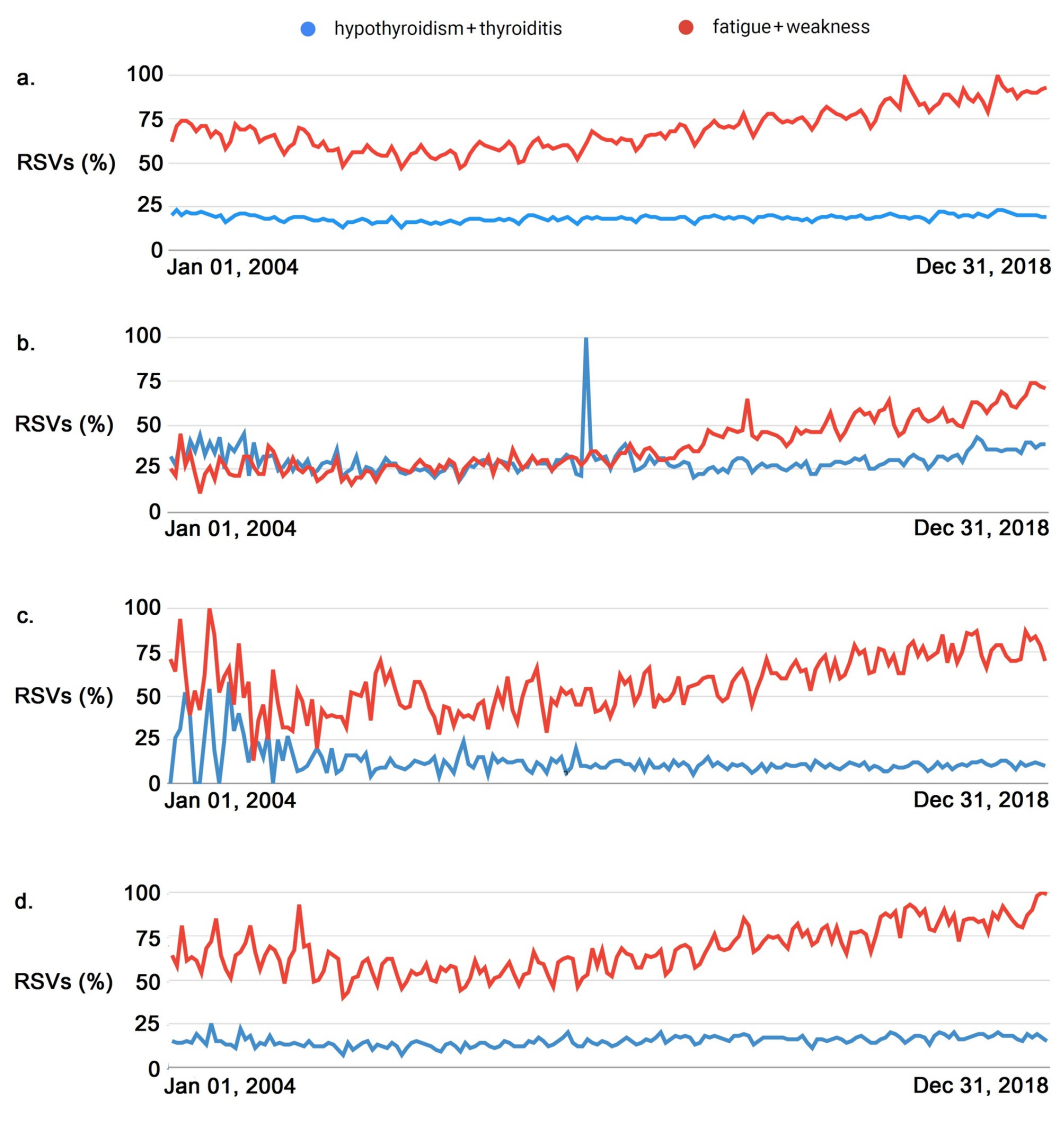
Time plots of relative [internet] search volumes (RSVs) for “hypothyroidism+thyroiditis” and “fatigue+weakness” worldwide (3a), and from the southern hemisphere in Brazil (3b), South Africa (3c) and Australia (3d). Note that in all the data the RSVs for “fatigue+weakness” outnumber those for “hypothyroidism+thyroiditis”. The spike in 2011 in 3b corresponds to a change in data gathering parameters by Google but is not noted in the other regions/countries. RSVs: Relative [internet] search volumes.

 Google’s output for long-term analysis was provided on a monthly basis and the results of RSVs are shown in Figures 1-3.

**Figure 4:**
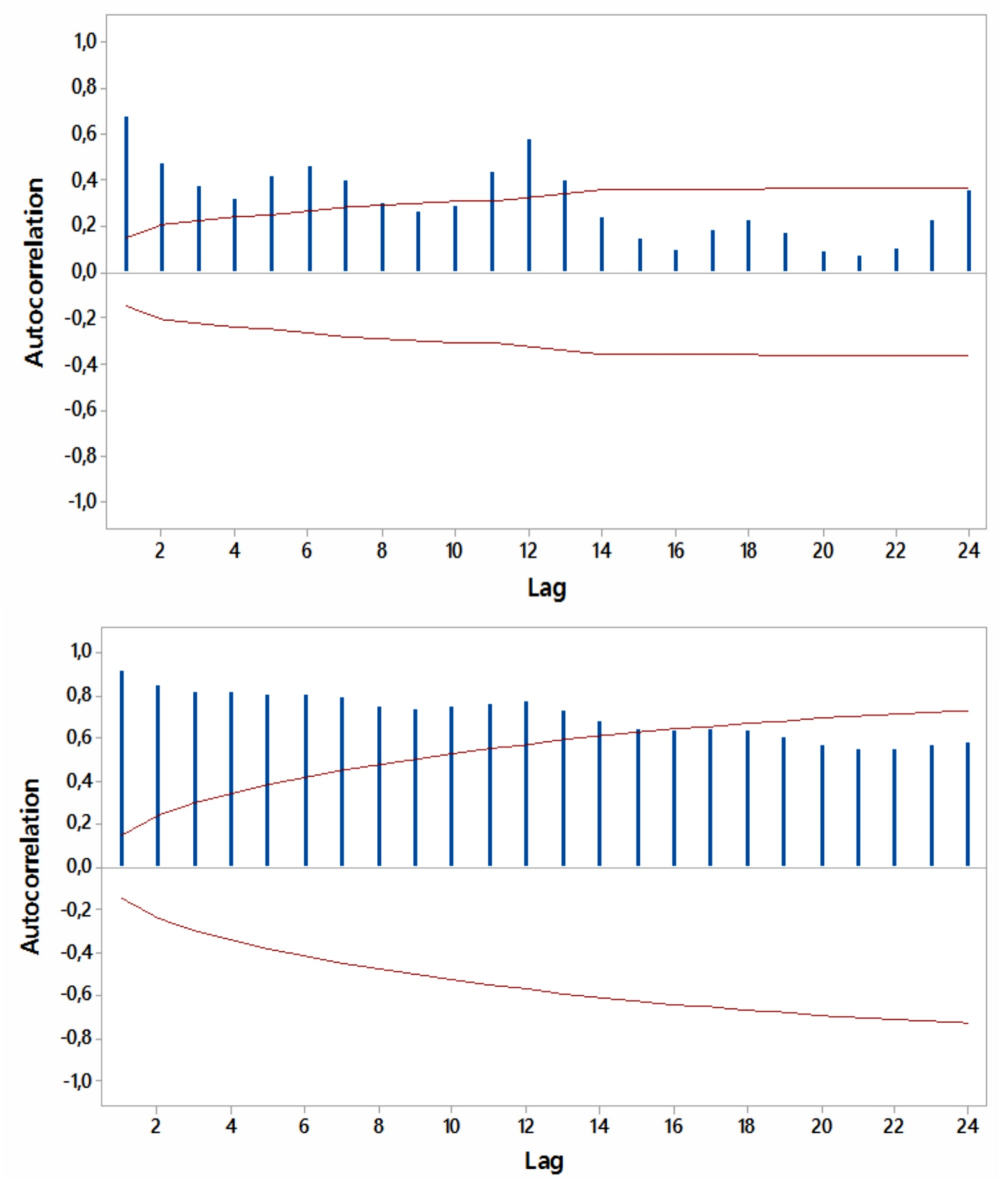
Autocorrelations for relative [internet] search volumes (RSVs) regarding “hypothyroidism+thyroiditis” (upper panel) and “fatigue+weakness” (lower panel) worldwide (in English) with 5% significance limits (in red); lag time in months.

**Figure 5:**
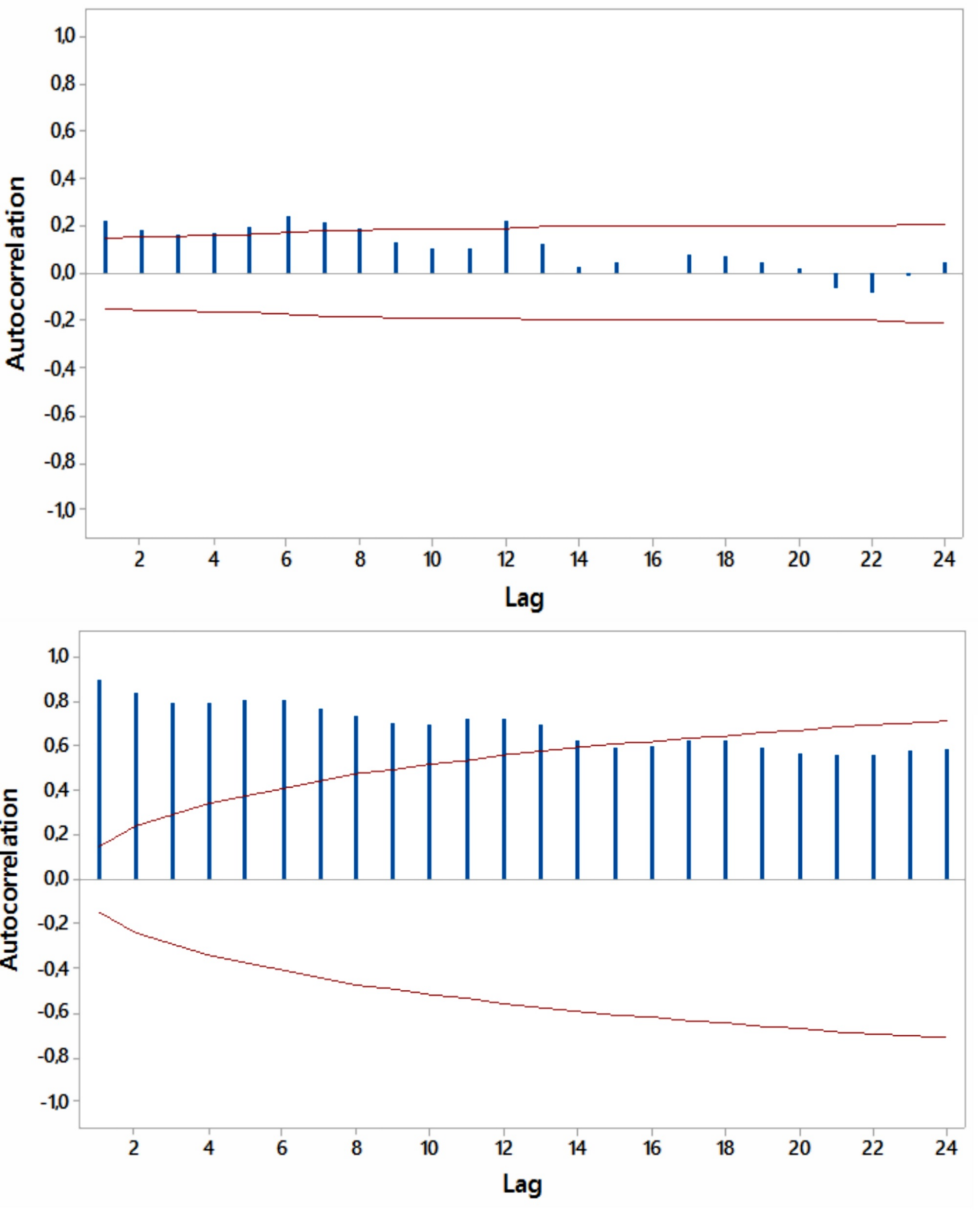
Autocorrelations for relative [internet] search volumes (RSVs) regarding “hypothyroidism+thyroiditis” (upper panel) and “fatigue+weakness” (lower panel) in Brazil (in Portuguese) with 5% significance limits (in red); lag time in months.

**Figure 6:**
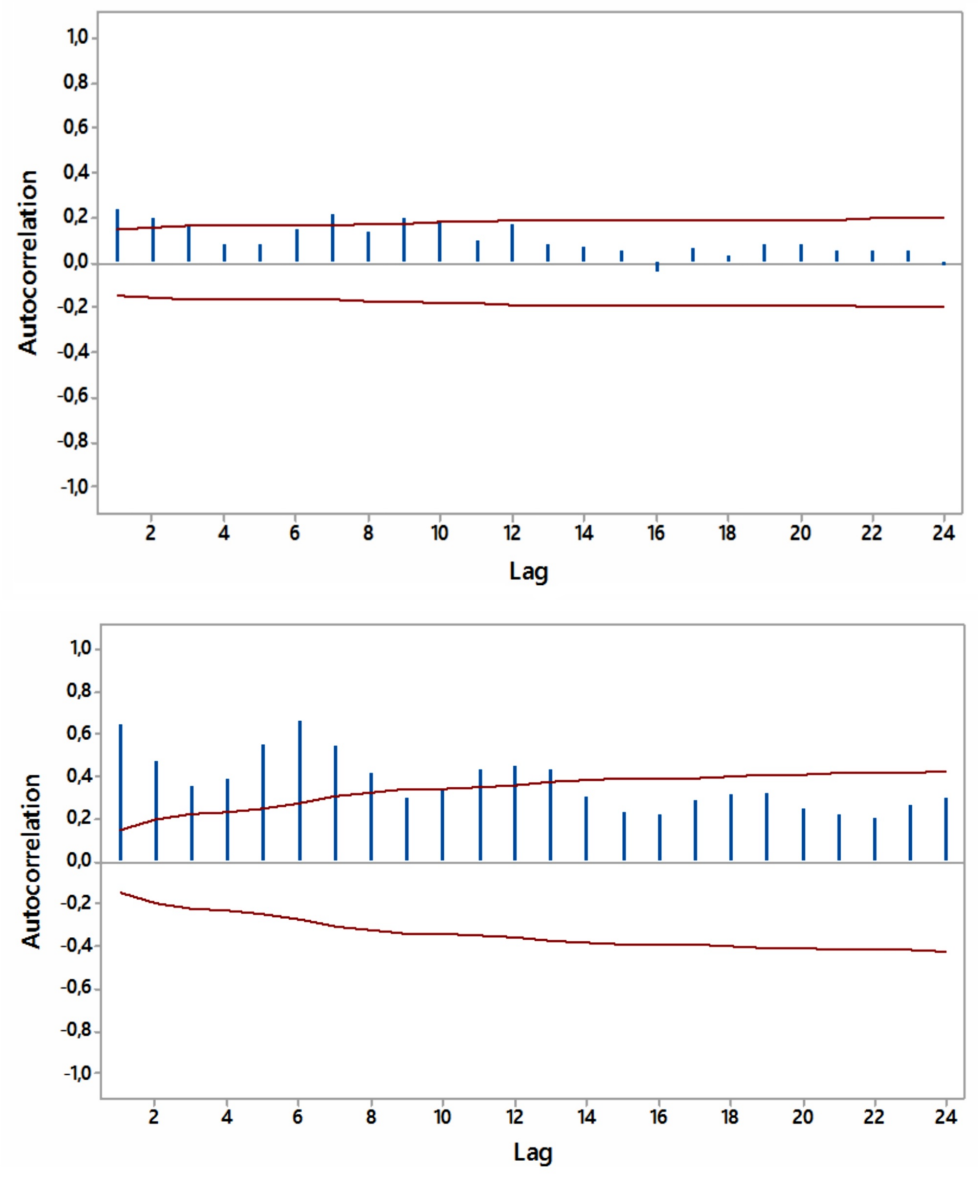
Autocorrelations for relative [internet] search volumes (RSVs) regarding “hypothyroidism+thyroiditis” (upper panel) and “fatigue+weakness” (lower panel) in South Africa (in English) with 5% significance limits (in red); lag time in months.

**Figure 7:**
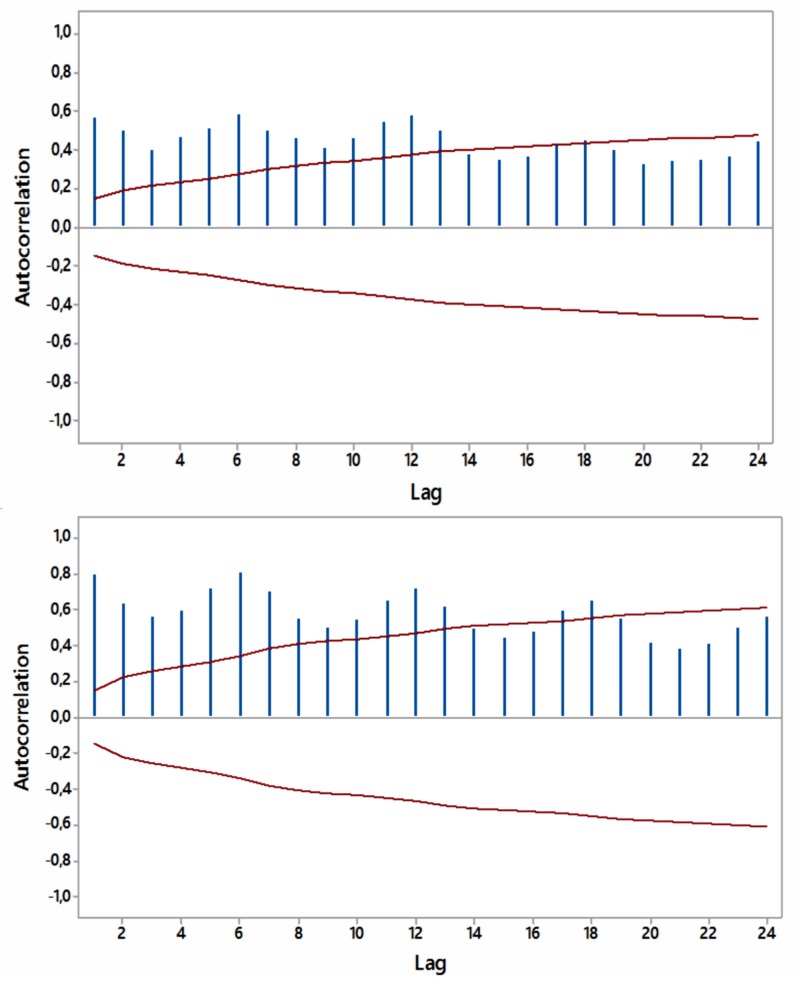
Autocorrelations for relative [internet] search volumes (RSVs) regarding “hypothyroidism+thyroiditis” (upper panel) and “fatigue+weakness” (lower panel) in Australia (in English) with 5% significance limits (in red); lag time in months.

Most ACFs were significant (p<0.05) within a year’s span, with peaks at six and 12 months, thus periodicity in the relevant internet searches was noted (Figures 4-7).

**Figure 8:**
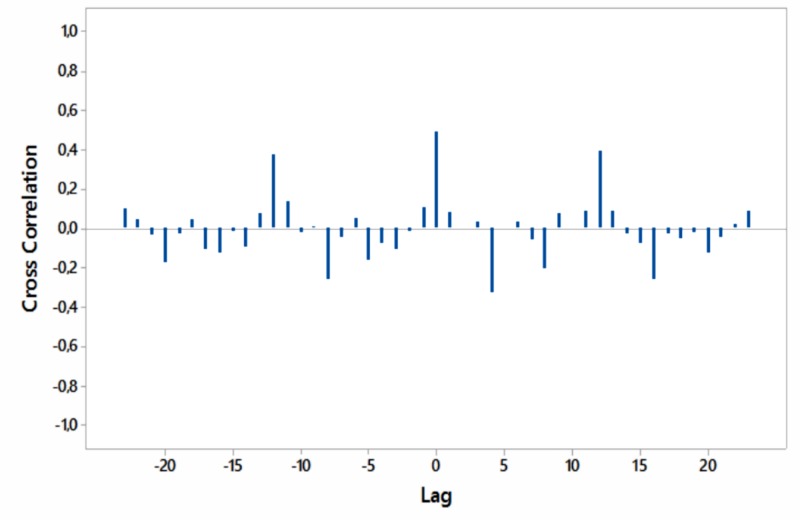
Cross-correlations of relative [internet] search volumes (RSVs) regarding “hypothyroidism+thyroiditis” vs RSVs regarding “fatigue+weakness” (in English) worldwide; lag time in months.

**Figure 9:**
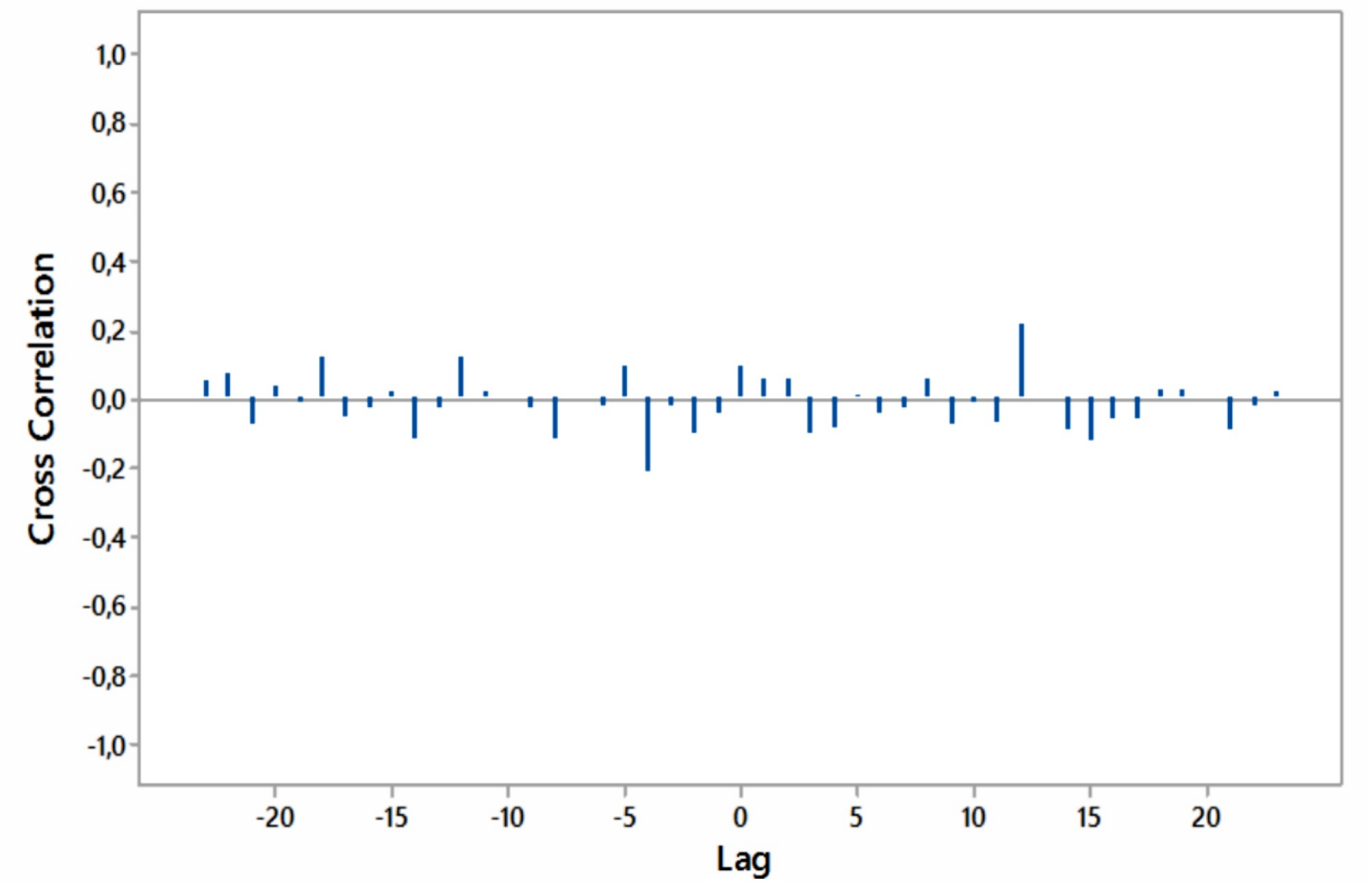
Cross-correlations of relative [internet] search volumes (RSVs) regarding “hypothyroidism+thyroiditis” vs RSVs regarding “fatigue+weakness” in Brazil (in Portuguese); lag time in months.

**Figure 10:**
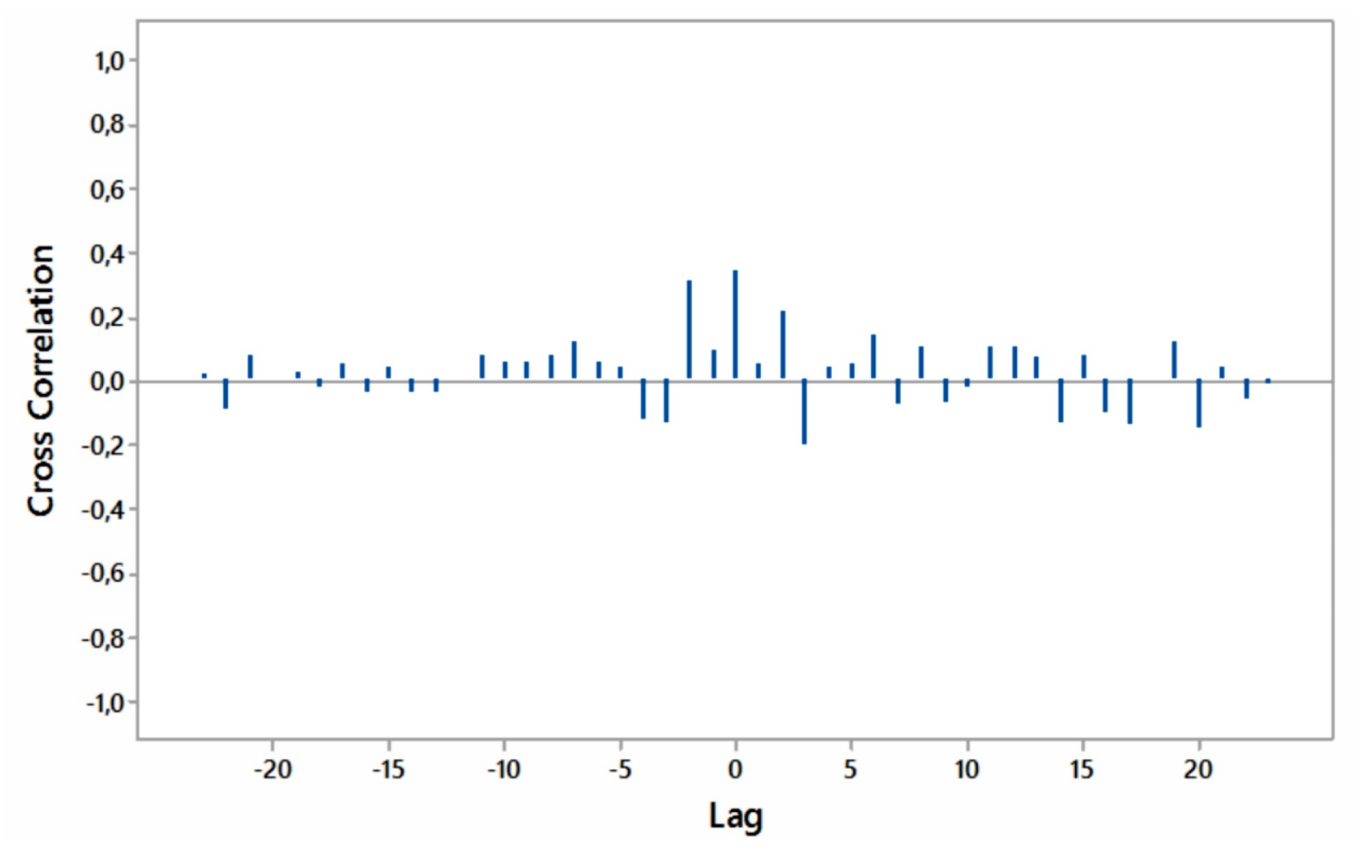
Cross-correlations of relative [internet] search volumes (RSVs) regarding “hypothyroidism+thyroiditis” vs RSVs regarding “fatigue+weakness” in South Africa (in English); lag time in months.

**Figure 11:**
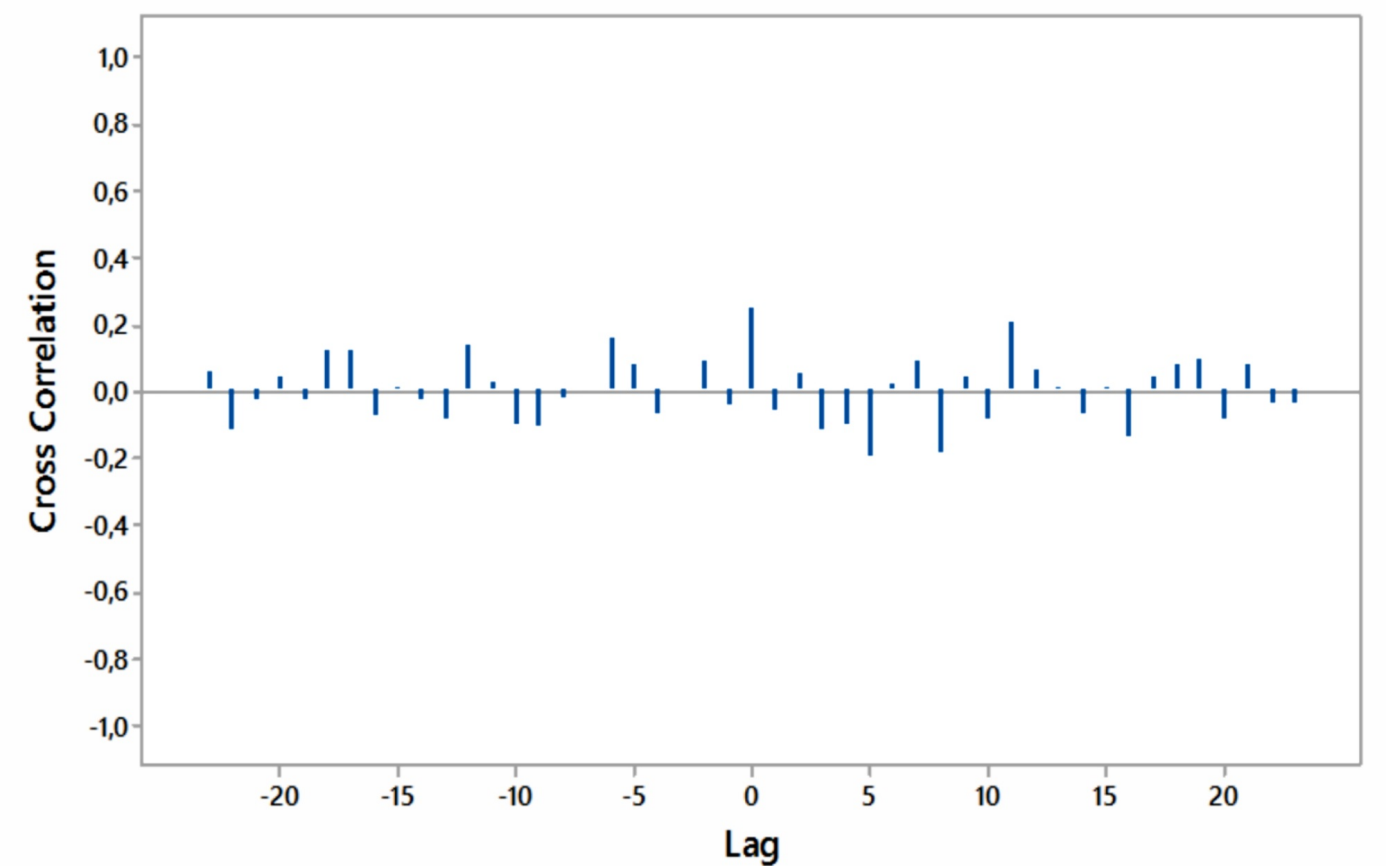
Cross-correlations of relative [internet] search volumes (RSVs) regarding “hypothyroidism+thyroiditis” vs RSVs regarding “fatigue+weakness” in Australia (in English); lag time in months.

Significant cross-correlations with no lag were noted between internet searches for “hypothyroidism+thyroiditis” and “fatigue+weakness” worldwide as well as for South Africa and Australia (Figures 8-11).

Worldwide there were significantly more RSVs for “hypothyroidism+thyroiditis” during the northern hemisphere’s spring (March, April, and May) (p<0.05, Table 1), whereas more RSVs were also noted in the southern hemisphere’s spring (September, October, and November) (with p<0.05, Tables 2-4).

**Table 1:** Post-hoc comparisons of relative [internet] search volumes (RSVs) by season worldwide (vis-à-vis northern hemisphere seasons). Kruskal-Wallis test statistic (K-W) = 17.278, p < 0.001 for “hypothyroidism+thyroiditis”, K-W = 8.055, p = 0.0447 for “fatigue+weakness”

Comparison of RSVs			
For “hypothyroidism+thyroiditis”			
Factor (Season)	n	Average Rank	Different (p<0.05) from factor
1 (Winter)	45	73.84	2
2 (Spring)	45	117.19	1, 3, 4
3 (Summer)	45	86.59	2
4 (Autumn)	45	84.38	2
For “fatigue+weakness”			
Factor (Season)	n	Average Rank	Different (p<0.05) from factor
1 (Winter)	45	73.58	2
2 (Spring)	45	104.44	1
3 (Summer)	45	92.59	
4 (Autumn)	45	91.39	

**Table 2:** Post-hoc comparisons of relative [internet] search volumes (RSVs) by southern hemisphere season for Brazil. Kruskal-Wallis test statistic (K-W) = 28.650, p < 0.001 for “hypothyroidism+thyroiditis”, K-W = 8.853, p = 0.031 for “fatigue+weakness”

Comparison of RSVs			
For “hypothyroidism+thyroiditis”			
Factor (Season)	n	Average Rank	Different (p<0.05) from factor
1 (Summer)	45	62.82	2, 4
2 (Autumn)	45	100.87	1, 3
3 (Winter)	45	80.38	2, 4
4 (Spring)	45	117.93	1, 3
For “fatigue+weakness”			
Factor (Season)	n	Average Rank	Different (p<0.05) from factor
1 (Summer)	45	82.41	4
2 (Autumn)	45	96.66	
3 (Winter)	45	76.82	4
4 (Spring)	45	106.11	1, 3

**Table 3:** Post-hoc comparisons of relative [internet] search volumes (RSVs) by southern hemisphere season for South Africa. Kruskal-Wallis test statistic (K-W) = 11.654, p = 0.008 for “hypothyroidism+thyroiditis”, K-W = 11.422, p = 0.009 for “fatigue+weakness”

Comparison of RSVs			
For “hypothyroidism+thyroiditis”			
Factor (Season)	n	Average Rank	Different (p<0.05) from factor
1 (Summer)	45	86.56	4
2 (Autumn)	45	84.32	4
3 (Winter)	45	78.27	4
4 (Spring)	45	112.86	1, 2, 3
For “fatigue+weakness”			
Factor (Season)	n	Average Rank	Different (p<0.05) from factor
1 (Summer)	45	79.97	4
2 (Autumn)	45	99.58	3
3 (Winter)	45	75.50	2, 4
4 (Spring)	45	106.96	1, 3

**Table 4:** Post-hoc comparisons of relative [internet] search volumes (RSVs) by southern hemisphere season for Australia. Kruskal-Wallis test statistic (K-W) = 13.998, p = 0.003 for “hypothyroidism+thyroiditis”, K-W = 19.594, p < 0.001 for “fatigue+weakness”

Comparison of RSVs			
For “hypothyroidism+thyroiditis”			
Factor (Season)	n	Average Rank	Different (p<0.05) from factor
1 (Summer)	45	74.81	4
2 (Autumn)	45	94.17	
3 (Winter)	45	80.50	4
4 (Spring)	45	112.52	1, 3
For “fatigue+weakness”			
Factor (Season)	n	Average Rank	Different (p<0.05) from factor
1 (Summer)	45	69.90	2, 4
2 (Autumn)	45	105.30	1, 3
3 (Winter)	45	77.24	2, 4
4 (Spring)	45	109.56	1, 3

## Discussion

Our study is based on the premise that health information-seeking behavior on the internet reflects disease epidemiology [12]. We searched for seasonality in internet searches for hypothyroidism worldwide (reflecting mainly the northern hemisphere, since 90% of the world’s population lives there [13]) as well as separately for selected countries in the southern hemisphere. Hypothyroidism and thyroiditis as well as the commonest hypothyroidism symptoms were more popular in internet searches at springtime in the northern and the southern hemispheres (we did not search for other symptoms of hypothyroidism because the search for other, albeit non-specific, complaints like “hair+loss” yielded RSVs so high that dwindled the RSVs for “hypothyroidism+thyroiditis” and did not permit any further analysis – data not shown for brevity). Thus, although this analysis is coarse, it seems that some seasonality (probably associated with the weather/climate or seasonal human activities) can be inferred on hypothyroidism. The latter may include disease as a result of autoimmune or subacute thyroiditis. In the literature, peak thyroid hormone levels (and indirectly less often hypothyroidism), have been reported during summer in longitudinal studies [3], while in a large study (n=11806) a small increase in free triiodothyronine (but not in thyrotropin or free thyroxine) was noted during winter [14].

We have to acknowledge the limitations of the study: biases regarding internet searches vis-à-vis a real diagnosis of hypothyroidism might exist and calendar effects (a statistically significant relationship between a particular time and a measured parameter) might also be implicated [15]. At least in the United States, 66% of those over 65 years of age use the internet, compared to 87% for those aged between 50-64 and 97% for those aged between 18 and 49 [16]. This gap in internet use by age group - which is apparent worldwide - potentially biases our study towards age groups that have access to the internet and are familiar with internet searches. Of course causation may not be inferred by the similar time patterns of thyroid disease and symptoms, and another limitation of the study is that apparent internet search traffic is also driven by media reports of scientific findings from peer-reviewed journals and scientific conferences [17]; such items were not taken into account in the present study. Thyroid disease awareness day worldwide is on May 25th, while January is set as thyroid disease awareness month in the United States by the American Thyroid Association. Although the effectiveness of such disease awareness days/months is debatable, they may influence relevant information-seeking on the internet [18]. Additionally, a higher percentage of the population may be concerned about additional weight in the spring and, as a result, looks up for thyroid diseases at that time, in view of the popular notion that thyroid problems often cause weight gain [19]. Finally, internet searches might not be appropriate for the study of chronic diseases such as hypothyroidism, as critics of internet-based research for various diseases suggest [4, 20].

## Conclusions

Thyroid diseases may show seasonality in their epidemiology. The latter may be reflected by health information-seeking behavior on the internet. We searched for seasonal patterns in internet searches (during 2004 - 2018) in the northern and southern hemispheres regarding hypothyroidism and thyroiditis (its commonest cause) as well for fatigue and weakness (the most prevalent symptoms of hypothyroidism). The internet searches for these thyroid diseases and their commonest symptoms were correlated worldwide and were more prevalent at springtime in both the northern and the southern hemispheres. Of course, more people may be concerned about their additional weight during springtime and, as a result, are more actively seeking information on the internet regarding thyroid diseases, based on the notion that hypothyroidism leads to weight gain. Nevertheless, despite the limitations of our approach it seems that some degree of seasonality can be inferred on hypothyroidism.
